# The Dynamic of Extracellular Vesicles in Patients With Subacute Stroke: Results of the “Biomarkers and Perfusion—Training-Induced Changes After Stroke” (*BAPTISe*) Study

**DOI:** 10.3389/fneur.2021.731013

**Published:** 2021-11-08

**Authors:** Ruben A. Jödicke, Shufan Huo, Nicolle Kränkel, Sophie K. Piper, Martin Ebinger, Ulf Landmesser, Agnes Flöel, Matthias Endres, Alexander H. Nave

**Affiliations:** ^1^Center for Stroke Research Berlin, Charité-Universitätsmedizin Berlin, Berlin, Germany; ^2^Klinik und Hochschulambulanz für Neurologie, Charité-Universitätsmedizin Berlin, Berlin, Germany; ^3^German Center for Cardiovascular Disease, Partner Site Berlin, Berlin, Germany; ^4^Klinik für Kardiologie, Charité-Universitätsmedizin Berlin, Berlin, Germany; ^5^NeuroCure Clinical Research Center, Charité Universitätsmedizin Berlin, Berlin, Germany; ^6^Medical Park Berlin Humboldtmühle, Berlin, Germany; ^7^Department of Neurology, University Medicine Greifswald, Greifswald, Germany; ^8^German Center for Neurodegenerative Diseases, Rostock/Greifswald, Germany; ^9^Berlin Institute of Health (BIH), Berlin, Germany; ^10^German Center for Neurodegenerative Disease, Partner Site Berlin, Berlin, Germany

**Keywords:** stroke, biomarker, extracellular vesicle (EV), subacute stroke, functional recovery

## Abstract

**Objective:** Extracellular vesicles (EV) are sub-1 μm bilayer lipid coated particles and have been shown play a role in long-term cardiovascular outcome after ischemic stroke. However, the dynamic change of EV after stroke and their implications for functional outcome have not yet been elucidated.

**Methods:** Serial blood samples from 110 subacute ischemic stroke patients enrolled in the prospective *BAPTISe* study were analyzed. All patients participated in the *PHYS-STROKE* trial and received 4-week aerobic training or relaxation sessions. Levels of endothelial-derived (EnV: Annexin V+, CD45–, CD41–, CD31+/CD144+/CD146+), leukocyte-derived (LV: Annexin V+, CD45+, CD41–), monocytic-derived (MoV: Annexin V+, CD41–, CD14+), neuronal-derived (NV: Annexin V+, CD41–, CD45–, CD31–, CD144–, CD146–, CD56+/CD171+/CD271+), and platelet-derived (PV: Annexin V+, CD41+) EV were assessed via fluorescence-activated cell sorting before and after the trial intervention. The levels of EV at baseline were dichotomized at the 75th percentile, with the EV levels at baseline above the 75th percentile classified as “high” otherwise as “low.” The dynamic of EV was classified based on the difference between baseline and post intervention, defining increases above the 75th percentile as “high increase” otherwise as “low increase.” Associations of baseline levels and change in EV concentrations with Barthel Index (BI) and cardiovascular events in the first 6 months post-stroke were analyzed using mixed model regression analyses and cox regression.

**Results:** Both before and after intervention PV formed the largest population of vesicles followed by NV and EnV. In mixed-model regression analyses, low NV [−8.57 (95% CI −15.53 to −1.57)] and low PV [−6.97 (95% CI −13.92 to −0.01)] at baseline were associated with lower BI in the first 6 months post-stroke. Patients with low increase in NV [8.69 (95% CI 2.08–15.34)] and LV [6.82 (95% CI 0.25–13.4)] were associated with reduced BI in the first 6 months post-stroke. Neither baseline vesicles nor their dynamic were associated with recurrent cardiovascular events.

**Conclusion:** This is the first report analyzing the concentration and the dynamic of EV regarding associations with functional outcome in patients with subacute stroke. Lower levels of PV and NV at baseline were associated with a worse functional outcome in the first 6 months post-stroke. Furthermore, an increase in NV and LV over time was associated with worse BI in the first 6 months post-stroke. Further investigation of the relationship between EV and their dynamic with functional outcome post-stroke are warranted.

**Clinical Trial Registration:** clinicaltrials.gov/, identifier: NCT01954797.

## Introduction

Stroke is the most frequent neurological cause for disability-adjusted life years (DALYs) worldwide (1). Long-lasting impairment in a patient's daily life is common after stroke. However, it is still difficult for clinicians to predict each patient's recovery potential and influencing factors (2). Clinical features account only for a portion of the predictable outcome (3, 4). Novel biomarkers are required aiming to adequately predict post-stroke recovery. These new biomarkers might also enable us to better understand the biological processes leading to clinical heterogeneity in stroke patients (5, 6).

The family of extracellular vesicles (EV) is a heterogeneous group of particles, formed by outward budding or exocytosis of the origin cell. Their diameter ranges between 100 nm and 1 μm. The outer membrane is formed by a lipid bilayer membrane that encloses the cargo of the respective particle (7). The concentration and differentiation of EV are potential biomarkers with a prognostic value. The association between EV and coronary artery disease is well-described, and studies have demonstrated involvement of EV in coagulation, inflammation, immune regulation, and angiogenesis, factors which also play a role in the pathomechanisms of stroke (8, 9).

Simak et al. showed in a cohort of 41 patients that levels of endothelial EV (EnV) after acute ischemic stroke are increased compared to a control group and correlate with stroke lesion size. Furthermore, higher levels of EnV in acute stroke were found in patients with moderate to severe stroke compared to mild stroke. In addition, high levels of EnV in acute stroke were associated with a lower Barthel Index (BI) at discharge (10). Jung et al. demonstrated an association of EnV to the national institute of health stroke scale (NIHSS) in acute stroke (11). Not only are EV potential biomarkers in the acute phase after stroke but, as Huo et al. demonstrated in a prospective cohort of 571 stroke patients, they also correlate with the number of recurrent cardiovascular events. They showed that high levels of EnV, leukocyte-derived EV (LV), and platelet-derived EV (PV) were associated with a worse cardiovascular outcome within 3 years (12). Levels of EV are not only increased in acute but also in subacute stroke as Lundström et al. demonstrated in a case–control study with healthy individuals. They also showed an association between increased tissue-factor bearing vesicles in acute stroke and cardiovascular events during follow up (13). Li and Qin demonstrated that EnV are also weakly correlated to the subtype of stroke in the Oxfordshire Community Stroke Project (OCSP) classification system (14). In a cohort of patients with coronary artery disease (CAD), van Craenbrook et al. demonstrated that over the course of 12 weeks aerobic exercise did not lead to an increase in EnV. However, patients with lower baseline concentrations of EnV showed a better functional response to the training sessions (15). Most of these studies investigated the levels of EV in the acute phase after stroke. The associations of dynamic changes in vesicles in subacute stroke and patient outcome have not been investigated in previous trials. Thus, we aimed to investigate the relationship between EV levels in subacute stroke and their dynamic changes with functional recovery and major vascular events up to 6 months after stroke.

## Methods

### The *BAPTISe*-Study

The “Biomarkers and Perfusion—Training-Induced changes after Stroke” study (*BAPTISe*, clinicaltrials.gov identifier: NCT01954797) is a prospective, endpoint-blinded longitudinal observational substudy of a randomized controlled trial named “Physical Fitness Training in Patients with Subacute Stroke” (*PHYS-STROKE*, clinicaltrials.gov identifier: NCT01953549). For further details we refer to the previously published study protocols (16, 17). Inclusion criteria for *BAPTISe* can be found in Supplementary Table 1. Here, we briefly summarize *PHYS-STROKE*: 200 patients with subacute and moderate to severe stroke were randomized to a fitness group performing 25 min of treadmill-based, aerobic fitness training five times a week for 4 weeks, or a relaxation group, performing muscle relaxation with the same duration and frequency. The trial did not a show a significant difference in the co-primary efficacy endpoints, maximal walking speed, and Barthel Index (BI) at 3 months post stroke, but a higher rate of serious adverse events in the fitness group; 110 patients of the *PHYS-STROKE* cohort participated in *BAPTISe*. All patients in *BAPTISE* suffered from subacute ischemic stroke (5–45 days after stroke onset), scored a BI of <65 points, and received cerebral MRI (18). The intervention was started when the patients were clinically able to perform the exercise demanded by their intervention arm.

**Figure 1:**
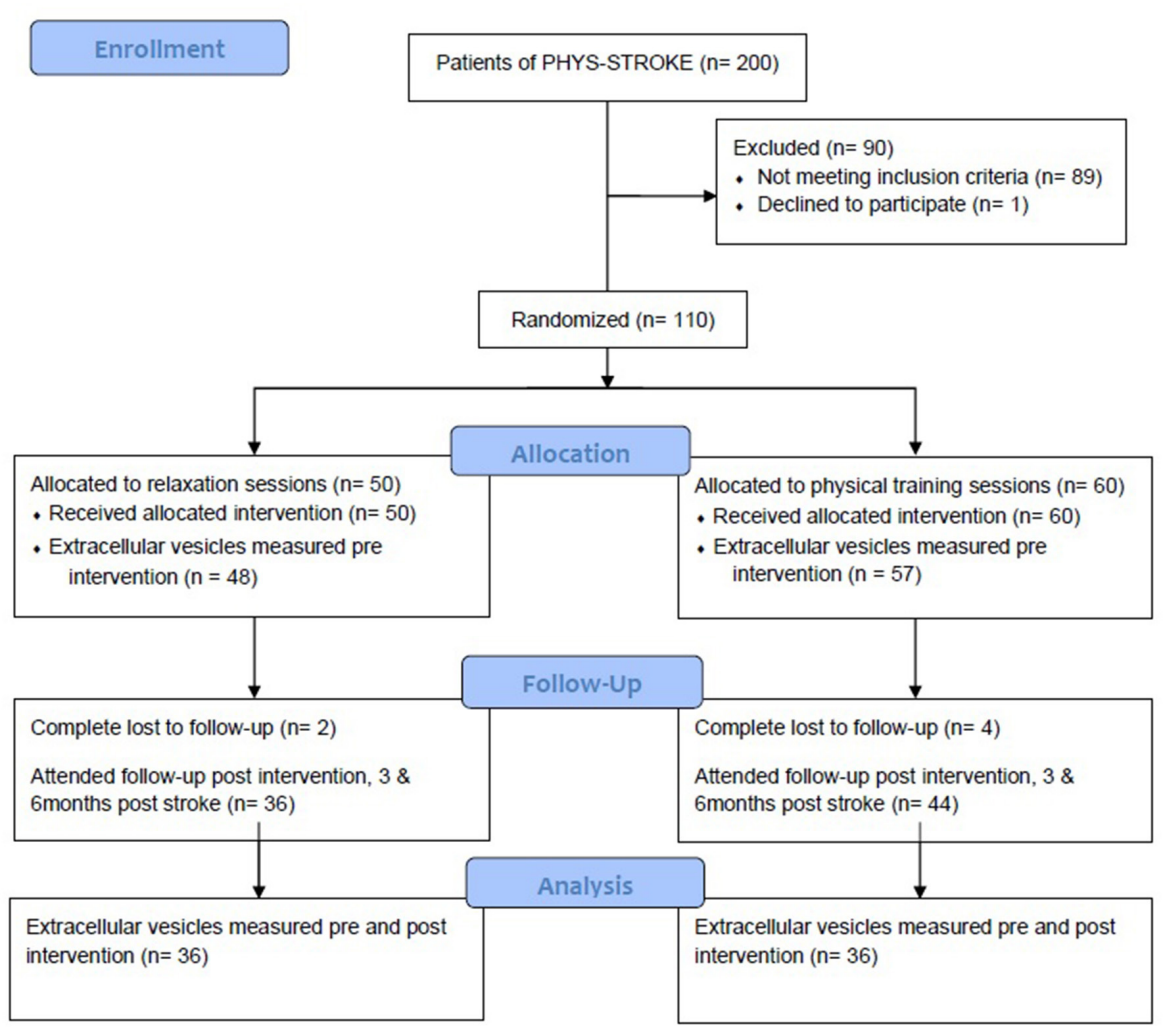
Consort flow diagram for the BAPTISe trial.

Levels of EV were measured before and after the intervention, which the patients underwent as part of *PHYS-STROKE*. The BI, a numerical score ranging from 0 to 100 ranking the patient higher for independence in activities of daily life, was evaluated pre-intervention, post-intervention, 3 months post-stroke, and 6 months post-stroke (see also Figure 1).

For this analysis, the BI, as a measure for functional outcome, was used as the primary outcome parameter. Our secondary outcome is the combined occurrence of major cardiovascular events being defined as ventricular flutter or fibrillation, myocardial infarction, recurrent stroke, or transient ischemic attack or death.

### Blood Draws and Measurement of Extracellular Microvesicles

We had drawn the blood samples shortly before the first day of intervention and shortly after the last day of the intervention by a study nurse. Blood samples of 4.5 ml were drawn and immediately centrifuged at 1,500×*g* for 15 min and 13,500×*g* for 5 min to retrieve platelet-free plasma and were subsequently stored at −80°C. The methodology for measurement of EV has been published previously (12). To summarize the essentials, quantification of EV concentrations was done using fluorescence-activated cell sorting (FACS). We used Attune Nxt acoustic focusing cytometer (Thermo Fisher Scientific, Carlsbad, CA, USA) with red (637 nm), blue (488 nm), and violet (405 nm) lasers to perform FACS. Our gating was performed with Kaluza Software (version 1.5; Beckman and Coulter, Brea, CA, USA). All EV were defined as Annexin V-binding particles and with a diameter below 1 μm. To define populations of EV we applied sequential gating to cell-surface specific markers (see Table 1).

**Table 1:** Definition of subpopulations of extracellular vesicles based on the surface markers.

**Origin cell**	**Surface markers**
All extracellular vesicles	Annexin V (AV)+
Platelet-derived EV	AV+ CD41+
Leukocyte-derived EV	AV+ CD45+
Monocyte-derived EV	AV+ CD45+ CD14+
Endothelial-derived EV	AV+ CD45– CD144+/CD146+/CD31+
Neuronal-derived EV	AV+ CD45– CD144– CD146– CD31– CD56+/CD171+/CD271+

### Statistical Analysis

Patients were dichotomized based on baseline EV levels, and dynamics in EV levels were defined as the difference between pre-intervention EV levels and post intervention EV-levels. In both cases, the cut-off was defined as the 75th percentile, to differentiate between high or low levels at baseline and high or low change in EVs. We used a mixed model approach for the change of BI depending upon EV levels and EV dynamics adjusting for BI pre-intervention, age, sex, NIHSS at baseline, and stroke etiology classified by TOAST (19). To model the occurrence of major cardiovascular events, we performed Cox proportional hazard analyses with adjustment for age, sex, NIHSS at baseline, and stroke etiology. The statistical analyses were predefined in an analysis plan. All analyses were performed using “R: A Language and Environment for Statistical Computing” version 3.6.2. For modeling the “lme4” package was used in version 1.1.23.

## Results

One hundred ten patients of the *PHYS-STROKE* study were included in the BAPTISe cohort and had levels of EV determined at least once. Concentrations of EV prior to intervention were obtained in 105 patients. In 82 patients EV were evaluated twice (before and after the trial intervention).

**Figure 2:**
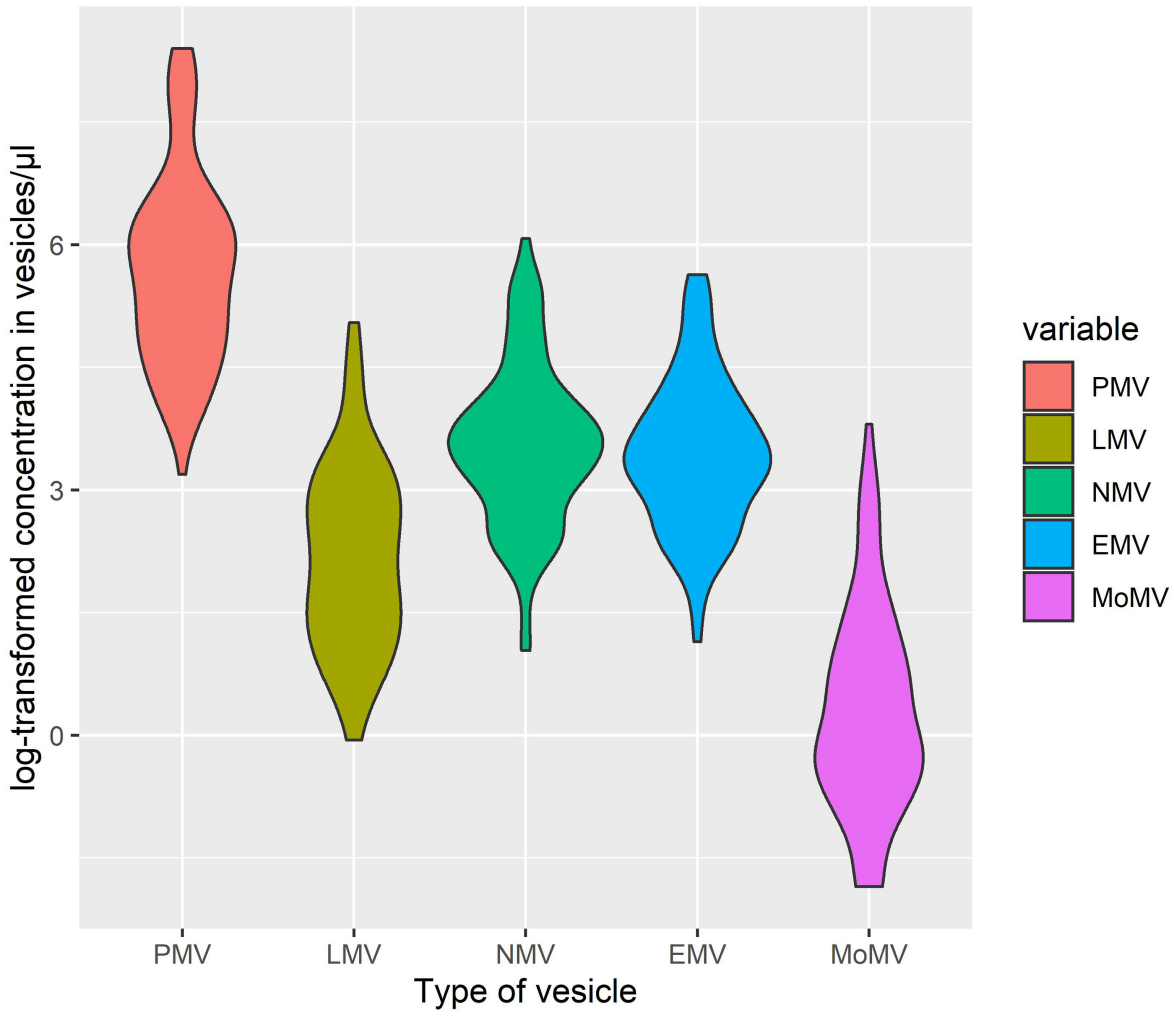
Violin-plot of log-transformed concentrations of extracellular vesicles subpopulations at baseline. PV, platelet-derived vesicles; LV, leukocyte-derived vesicles; NV, neuronal-derived vesicles; EnV, endothelial-derived vesicles; MoV, monocytal-derived vesicles.

Baseline characteristics including levels of EV of all patients are shown in Table 2. Our patient collective has a median age of 69, consists of ~41% females, and presented with a mean NIHSS of 9 on admission. There were no differences in BI or NIHSS at baseline regarding baseline EV-populations detected by Student's *t*-test (see Supplementary Table 2). The largest population of EV at baseline was formed by PV with a mean of 616.1/μl, followed by NV (mean: 56.9/μl), EnV (mean: 47.8/μl), LV (mean: 17.1/μl), and monocyte-derived EV (MoV) (mean: 3.2/μl) (see also Figure 2). After the intervention, the vesicle populations were unchanged in order and magnitude with PV forming the largest population (mean: 505.8/μl), followed by NV (mean: 52.8/μl), EnV (mean: 45.8/μl), LV (mean: 16.6/μl), and MoV (mean: 2.8/μl). Student's *t*-test did not reveal any difference between the distributions of vesicle concentration at baseline and at follow-up in each EV subtype (see Supplementary Figure 1). In addition, neither at baseline nor at follow-up, Students *t*-test detects any sex-related or intervention related differences in each vesicle population. We also did not find a correlation between NIHSS at admission and EV concentrations, neither at baseline nor at follow-up. We could also not detect an association of concentration in EV at baseline and time since stroke onset.

**Table 2:** Baseline characteristics of the full *BAPTISe* population.

**Variable**	**Baseline cohort** **(*n* = 110)**
Age in years (median, IQR)	69, 60–78.75
Sex (female, %)	45, 40.9
NIHSS at admission (median, IQR)	9, 6–12.75
Barthel Index at admission (median, IQR)	50, 35–60
Platelet vesicles pre-intervention in Vesicles/μl (mean **±** standard deviation)	616.09 ± 933.42
Neuronal vesicles pre-intervention in Vesicles/μl (mean **±** standard deviation)	56.91 ± 69.42
Endothelial vesicles pre-intervention in Vesicles/μl (mean **±** standard deviation)	47.84 ± 53.62
Leukocyte vesicles pre-intervention in Vesicles/μl (mean **±** standard deviation)	17.1 ± 25.83
Monocyte vesicles pre-intervention in Vesicles/μl (mean **±** standard deviation)	3.24 ± 6.23

The average Barthel Index showed an increase from 50 at baseline, up to a median of 90 at 6 months post-stroke (see also Supplementary Figure 2). We recorded 24 major cardiovascular events with a median time to event of 60 days in 37.4 person-years of follow-up (see also Table 3).

**Table 3:** Functional outcome parameters (BI, Barthel Index; IQR, interquartile range).

BI pre-intervention (median, IQR)	50, 35–60
BI post-intervention (median, IQR)	75, 55–90
BI at 3-months follow up (median, IQR)	80, 65–95
BI at 6-months follow up (median, IQR)	90, 70–100
Number of major events	24
Time to major events in days (median, IQR)	60, 41.5–98.25

**Figure 3:**
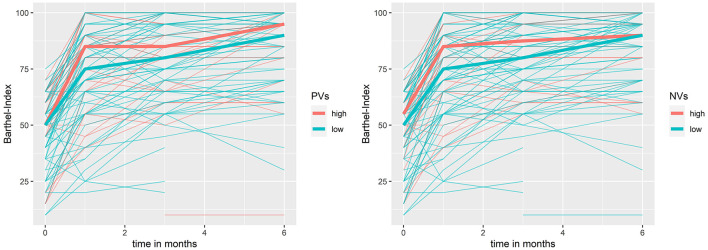
Barthel Index in the first 6 months post stroke by neuronal- and platelet-derived extracellular vesicles at baseline. bold line = median; NV, neuronal-derived vesicles at baseline; PV, platelet-derived vesicles at baseline.

**Figure 4:**
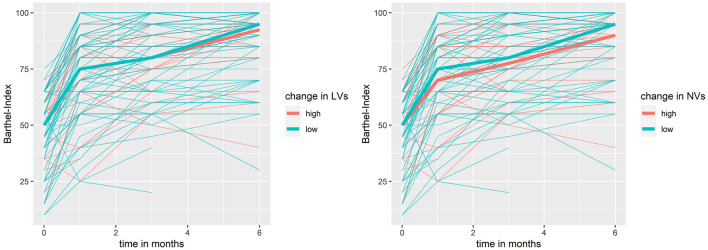
Barthel-Index in the first 6 months post stroke by dynamic in neuronal- and leukocyte-derived extracellular vesicles. bold line = median; NV, neuronal-derived vesicles; LV, leukocyte-derived vesicles.

Using mixed models with the BI in the first 6 months post-stroke as dependent and EV levels as independent variable, low PV levels and low NV levels at baseline were associated with lower BI values in the first 6 months post-stroke. For low PV at baseline, we observed a change in BI of −6.97 (95% CI: −15.53 to −1.6) and for low NV at baseline, a change in BI of −8.57 (95% CI: −13.92 to −0.01) in the first 6 months post-stroke (see Figure 3). Furthermore, we observed an association of an increase in LV and NV and a reduced BI in the first 6 months post-stroke, meaning that in patients that did not show an increase in LV, the BI in the 6 months post-stroke was higher by 6.82 (95% CI: 0.25–13.4). Patients which did not increase in NV showed an increase in BI in the 6 months post-stroke by 8.7 (95% CI: 2.1–15.34, see Figure 4). In Table 4, the effect of each vesicle at baseline and their dynamic on the BI can be found.

**Table 4:** Adjusted effect on the Barthel Index and the 95% Confidence interval of each vesicle at baseline and their dynamic from the mixed model.

**Vesicle type**	**Estimate of change in barthel index progression**	**95% Confidence interval**
Endothelial vesicles low	−6.97	−13.98 to 0.07
Leukocyte vesicles low	−4.35	−11.41 to 2.69
Monocyte vesicles low	−3.91	−11.14 to 3.34
Neuronal vesicles low	−8.57^*^	−15.53 to −1.6
Platelet vesicles low	−6.97^*^	−13.92 to −0.01
Endothelial vesicles low increase	6.77	−0.03 to 13.59
Leucocyte vesicles low increase	6.82^*^	0.25–13.4
Monocyte vesicles low increase	2.85	−3.82 to 9.46
Neuronal vesicles low increase	8.7^*^	2.1–15.34
Platelet vesicles low increase	4.58	−2.43 to 11.57

* *p < 0.05, adjusted for age, sex, baseline NIHSS, and TOAST*.

## Discussion

Our study showed that lower levels of NV and PV at baseline are associated with poor functional outcome in the first 6 months post-stroke. Furthermore, we could demonstrate an association in the patients with the largest increase in LV and NV in the subacute phase after stroke and a decreased BI in the first 6 months post-stroke. Neither the baseline levels of EV nor their change showed an association to cardiovascular risk and death 6 months post-stroke.

A previous systematic review by Wang and colleagues showed that various populations of EV are increased after ischemic stroke (20). One study could also demonstrate that EnV-levels assessed in the first 7 days post stroke were associated with stroke severity (14). In our report, we could not link baseline levels of EV to NIHSS or BI on admission (see Supplementary Table 2). However, Li and Qin detected differences in EV concentration in patients with NIHSS <5 and ≥5. Our patients, on the contrary, presented with a median NIHSS of 9 suffering from a more severe clinical syndrome. One possible interpretation would be that EV in acute stroke increase up to a certain threshold based on stroke severity and reach a ceiling effect.

We detected an association of low NV, PV, and EnV levels at baseline with a lower BI during the first 6 months post-stroke. In contrast, Simak and colleagues showed that in acute stroke, high levels of EnV are associated with a worse BI at hospital discharge (10). However, there are two key differences in the trials. In *BAPTISe*, we evaluated levels of EV in subacute stroke, ranging from day 5–45 post-stroke, whilst Simak et al. evaluated the levels of EV far earlier, with a median time of 37 h after symptom onset. Therefore, we are comparing the EV of patients in subacute stroke with patients in acute stroke. Furthermore, we evaluated BI at multiple predefined time-points with longer follow-up and not at hospital discharge. This reduces bias quite a bit since hospital discharge typically happens after clinical improvement in the patient which most likely is accompanied by improvement in BI. We also want to stress the point that the EnV were differently defined in the studies leading to measurement and comparison of different EV-populations. Furthermore, we want to emphasize that also in the same vesicle populations, paradoxical observations can be noted (21). In contrast to Jung et al. we could not demonstrate an association between EnV and NIHSS at admission (11). However, they obtained all their data on vesicles in the first 6 days post stroke in contrast to our 5–45 days post stroke. As the colleagues also demonstrated in their paper, the concentrations of EnV in their trial highly are dependent on the time since symptom onset, with concentrations being negatively correlated to the time since onset. Since such a correlation could not be found in our patients with subacute stroke, the concentration of EnV might reach a plateau. This is also underlined by the findings of Chiva–Blanch, in which they also did not find a change in EnV concentrations in subacute stroke (22).

In previous trials, PV in acute stroke were linked to small and large vessel occlusion (23, 24). Elevation of PV has been shown to be associated with a thrombotic state and activated platelets (25). However, we find that elevated levels of PV, in subacute stroke, are associated with a better BI post stroke. As has been shown, PV can also exhibit anticoagulatory abilities (26). Further research is needed to understand the underlying mechanisms of PV, especially in explaining the dichotomous effects they can exhibit. For future research, the comparison of tissue factor-positive and -negative PV populations promises interesting findings, as already demonstrated by Lundström et al. (13).

There are only few studies investigating the role of EV as biomarkers for long-term outcome in stroke patients. Huo and colleagues investigated EV levels in a cohort of 621 patients in acute stroke (the acute phase defined as the first week after stroke). They compared patients based on quartiles of EV levels and found an association of high EnV and LV levels and cardiovascular events or death over a follow-up of 3 years (12). Lee and colleagues on the other hand investigated EV levels in 298 patients in subacute stroke, defined as stroke in the last 3 months. They dichotomized their cohort for EnV levels at the 50th percentile and could also find an association of cardiovascular events or recurrent stroke over the course of 3 years (27). We were not able to replicate these findings in our trial. This is likely attributable to the smaller sample size and a way shorter follow-up time of 6 months compared to the two other studies. In addition, Lundström and colleagues showed the association of phosphatidyl-serine-negative PV which were also associated with recurrent ischemic stroke or myocardial infarction (13). This is a vesicle population, which we did not measure. However, this emphasizes the broad spectrum of EV types and possible mechanisms influencing the patients' follow-up.

All estimated Cox proportional hazards ratios and their 95% confidence intervals are listed in Table 5. The full presentation of corresponding Kaplan–Meier plots can be found in Supplementary Figures 3, 4.

**Table 5:** Adjusted proportional hazard ratio of cox regression and 95% confidence interval for vesicles at baseline and their dynamic for the occurrence of major cardiovascular events.

**Vesicle type**	**Hazard ratio estimate**	**95% Confidence interval**
Endothelial vesicles low	0.54	0.18–1.58
Leukocyte vesicles low	0.89	0.3–2.61
Monocyte vesicles low	0.89	0.3–2.68
Neuronal vesicles low	0.48	0.17–1.35
Platelet vesicles low	0.96	0.33–2.79
Endothelial vesicles low increase	1.55	0.28–8.68
Leucocyte vesicles low increase	1.49	0.28–8.01
Monocyte vesicles low increase	1.02	0.2–5.16
Neuronal vesicles low increase	1.44	0.27–7.77
Platelet vesicles low increase	4.85	0.54–43.61

*Adjusted for age, sex, baseline NIHSS, and TOAST*.

It has been demonstrated that CD34+, CD56+/Annexin V+ EV, labeled neural progenitor cell-derived vesicles, are also increased over a prolonged period post-stroke compared to patients matched for cardiovascular risk (22), which the authors interpreted as an indicator for breakdown of blood–brain barrier. The increase in LV could be interpreted as an inflammatory state (21), which has been associated with worse outcome in stroke (28). It has to be noted that we gated our NV differently and used surface markers that are associated with neuronal cells. Furthermore, we excluded all EV bearing typical surface antigens for other cell-types. However, it is not clear if the measured EV truly originate from neuronal cells, especially since the density of surface proteins on EV is different from their origin cell (29). Therefore, we want to emphasize that the quantification of NV is still experimental and not fully validated. However, it should be the goal of further research to validate the NV and investigate their role in stroke and other neurological diseases.

One of the main limitations of our study is the relatively short follow-up time. In addition, there are always subpopulations of EV, which were not investigated such as tissue-factor bearing vesicles or phosphatidyl-serine negative vesicles. A correction for confounding agents such as anticoagulation has also not been done. Also, the evaluation of NV in patient samples is still experimental and not validated. It is not clear if the measured EV are generated in the central nervous system and pass through the blood–brain barrier or are generated in the periphery. Furthermore, we are observing wide confidence intervals, giving our results less precision. A methodological weakness in our study is the single use of FACS for quantification and differentiation of EV and the omission of complementary resources. One should also note that even though our patients received two different interventions, no single-arm analysis was performed. This is mainly due to *PHYS-STROKE* not finding any effect of the intervention on BI but also due to resulting loss of power (18). It is, however, not clear if the intervention might have altered the results, e.g., having an effect on vesicle concentrations or modifying the event-rate for cardiovascular events. Therefore, we want to emphasize that in future research, different interventions and their effect on EV should be evaluated. As a final remark, we want to stress that in our study, no control group is given, which would give more context to the baseline levels and the dynamic of our EV.

The main strength on the other hand is sampling EV at two time points and having multiple follow-up visits regarding functional capacity of our patients at predefined time points. We were the first to link changes in vesicle population to functional outcome. In our study, we showed that both an increase in LV, which might be interpreted as in increase in inflammation, and an increase in NV are associated with a worse BI at follow-up. However, further studies are needed to investigate the mechanisms at place and to validate cut-offs for vesicles as prognostic tools. After better understanding EV, they might also be used as a therapeutic agent or target in the future.

## Data Availability Statement

The pseudonymized raw data will be made available upon reasonable request to the authors.

## Ethics Statement

The studies involving human participants were reviewed and approved by Ethics Commission of the Charité, EA1/137/13. The patients/participants provided their written informed consent to participate in this study.

## Author Contributions

AF, MEb, and AN conceived or designed and supervised the study. SH and NK designed the protocol for measuring vesicles. RJ drafted the manuscript and responsible for analyzing the data. SP contributed to statistical analysis. AF, MEb, and MEn obtained funding. All authors critically revised the manuscript for important intellectual content and gave final approval of the version to be published.

## Funding

This trial was supported by the German Ministry for Health and Education (01EO0801) through the Center for Stroke Research Berlin grant G.2.15. The funder had no role in study design, data collection, analysis or interpretation, or writing of the manuscript. AN was a participant in the BIH-Charité Clinician Scientist Program funded by the Charité –Universitätsmedizin Berlin and the Berlin Institute of Health. MEn received funding from DFG under Germany's Excellence Strategy—EXC-2049-−390688087, BMBF, DZNE, DZHK, EU, Corona Foundation, and Fondation Leducq.

## Conflict of Interest

ME reports grants rom Bayer and fees paid to the Charité from AstraZeneca, Bayer, Boehringer Ingelheim, BMS, Daiichi Sankyo, Amgen, GSK, Sanofi, Covidien, Novartis, and Pfizer, all outside the submitted work. The remaining authors declare that the research was conducted in the absence of any commercial or financial relationships that could be construed as a potential conflict of interest.

## Publisher's Note

All claims expressed in this article are solely those of the authors and do not necessarily represent those of their affiliated organizations, or those of the publisher, the editors and the reviewers. Any product that may be evaluated in this article, or claim that may be made by its manufacturer, is not guaranteed or endorsed by the publisher.
