# Supplementary material for: The Dynamic of Extracellular Vesicles in Patients With Subacute Stroke: Results of the “Biomarkers and Perfusion—Training-Induced Changes After Stroke” (BAPTISe) Study
Source: Front Neurol. 2021 Nov 8;12:731013. doi: 10.3389/fneur.2021.731013 (PMC8606784; doi:10.3389/fneur.2021.731013)
Supplement: Supplementary file 1 [file Data_Sheet_1.docx]

Supplementary Material

#
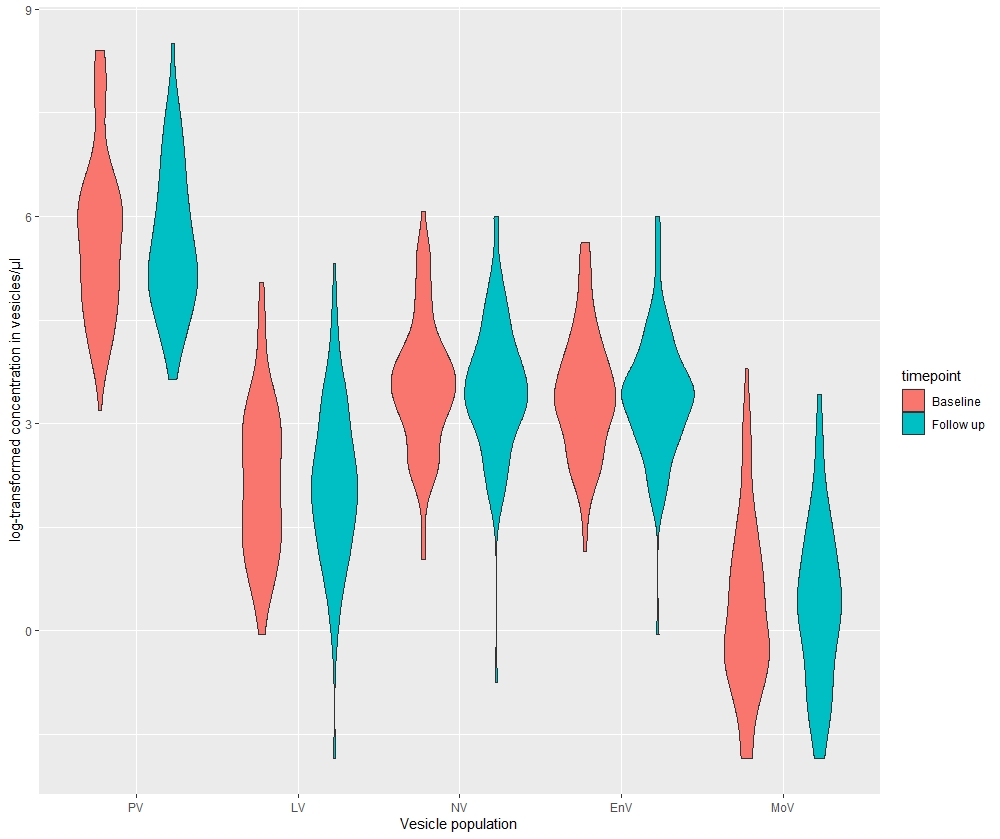
Supplementary Figures

supplementary Figure 1 – log-transformed concentrations of extracellular vesicles compared between pre- and postintervention, (PV = platelet-derived vesicles, LV = leucocyte-derived vesicles, NV = neuronal-derived vesicles, EnV = endothelial-derived vesicles, MoV = monocytal-derived vesicles)


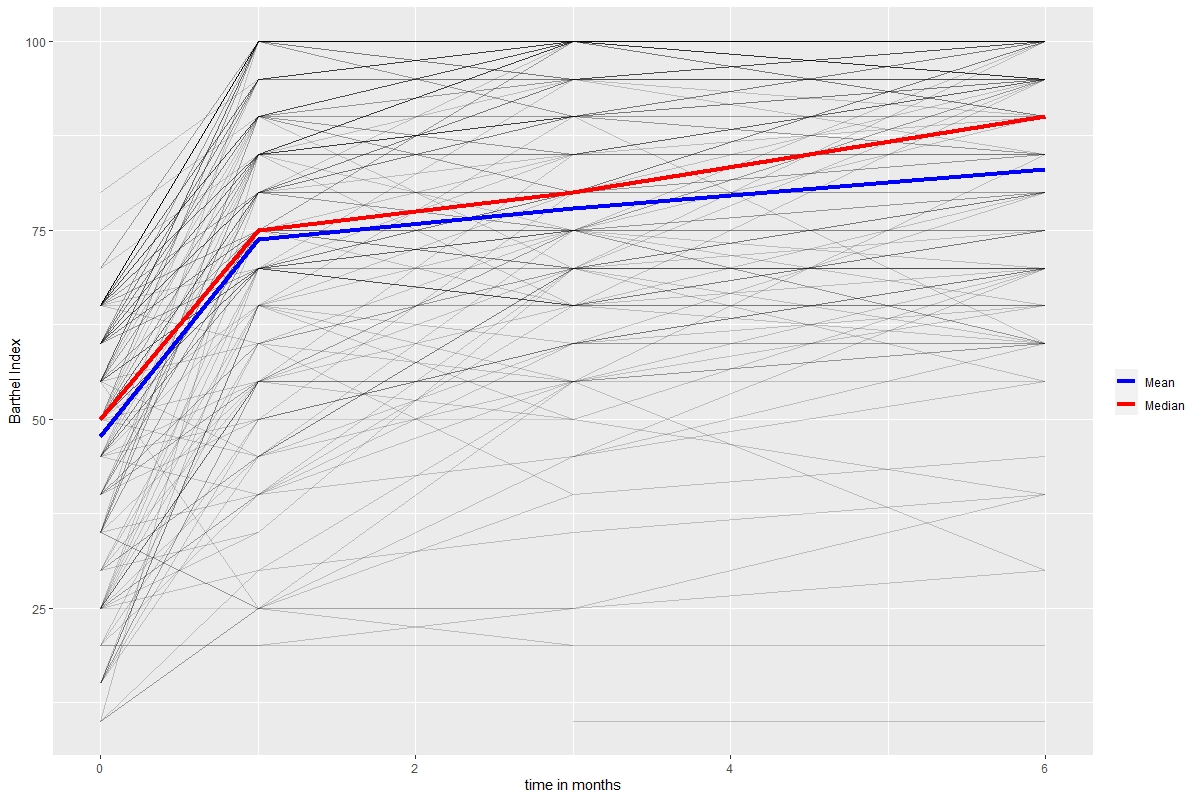


supplementary Figure 2 - Barthel-Index for each individual over 6 months post stroke, (red = Median, blue = Mean)

#
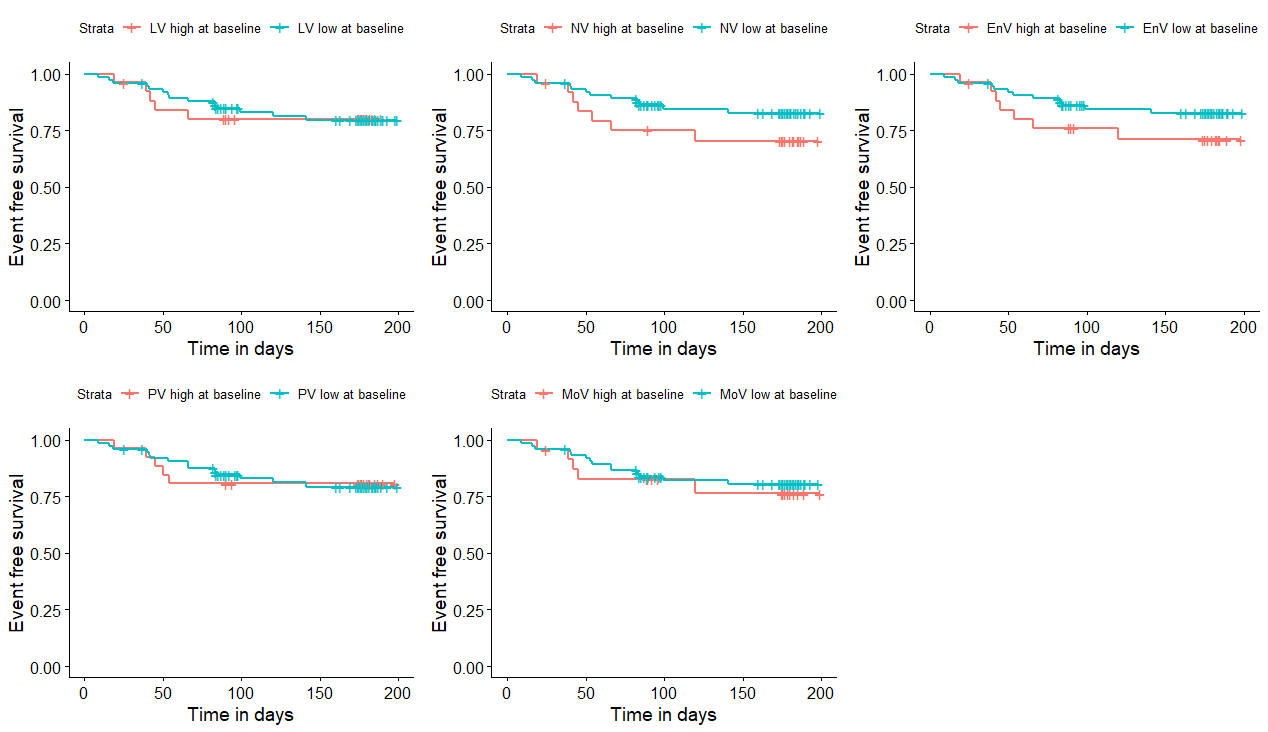


supplementary Figure 3 - Kaplan-Meier curves of event free survival discriminated by vesicle concentration at baseline, PV = platelet-derived vesicles, LV = leucocyte-derived vesicles, NV = neuronal-derived vesicles, EnV = endothelial-derived vesicles, MoV = monocytal-derived vesicles, (red = above 75th percentile, blue = below 75th percentile)

T
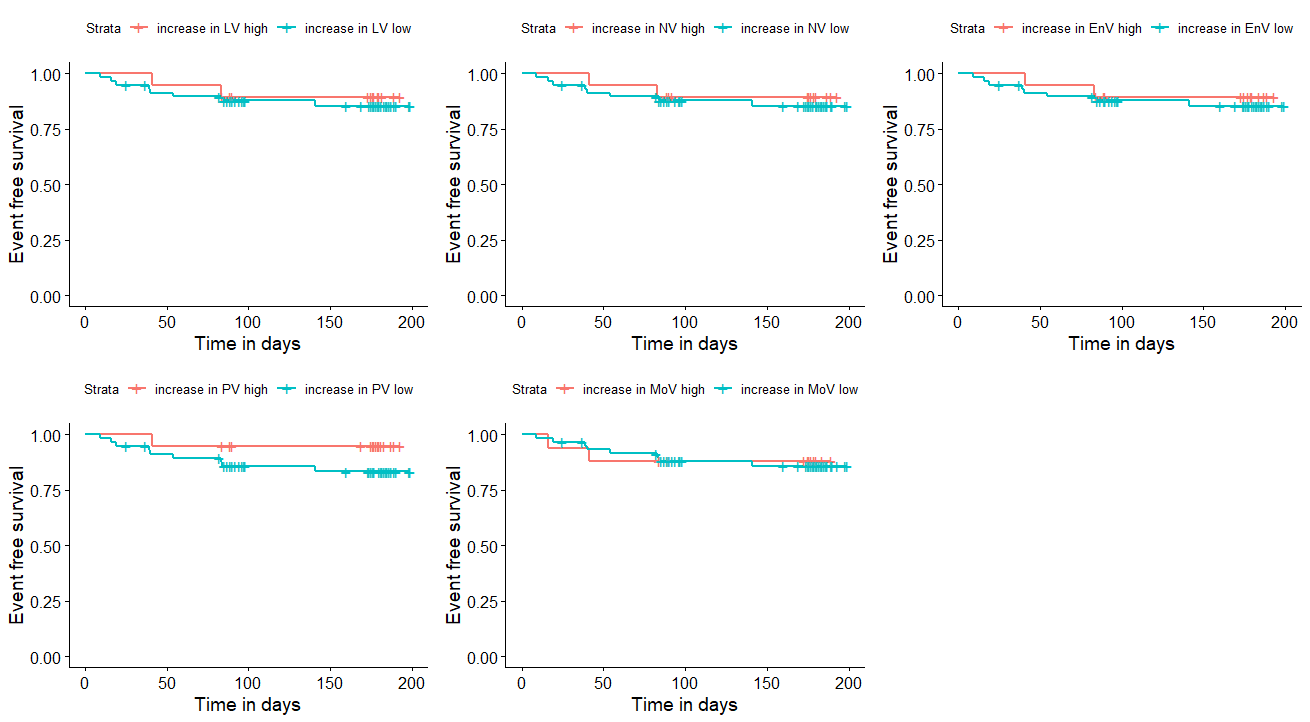


supplementary Figure 4 - Kaplan-Meier curves of event free survival discriminated by vesicle dynamic from pre- to post-intervention, PV = platelet-derived vesicles, LV = leucocyte-derived vesicles, NV = neuronal-derived vesicles, EnV = endothelial-derived vesicles, MoV = monocytal-derived vesicles, (red = above 75th percentile, blue = below 75th percentile)

# Supplementary Tables

supplementary Table 1 - Inclusion and Exclusion Criteria for BAPTISe (NCT: NCT01954797)

| Inclusion Criteria: |
| --- |
| - Age: > 18 years |
| - Diagnosis of subacute ischemic stroke (within 5-45 days after stroke onset)as determined by initial MRI/CT scan of the brain |
| - Cortical, sub-cortical, or brainstem affection |
| - Barthel Index (BI) <65 at inclusion |
| - Able to sit for at least 30 seconds (unsupported or supported, i.e., holding onto supports such as the edge of the bed) |
| - Ability to perform aerobic exercise, determined by by responsible physician |
| - Provision of written consent |
| Exclusion criteria: |
| - Lacking ability to comply with study requirements |
| - Stroke due to intracranial haemorrhage |
| - Previous subarachnoid hemorrhage or other hemorrhagic stroke |
| - Progressive stroke |
| - Not able to receive magnetic resonance imaging scans, including perfusion imaging |
| - Unable to perform the required exercises due to medical, musculoskeletal, or neurological problems |
| - Required help of at least 1 person to walk before stroke due to neurological (e. g., advanced Parkinson's disease, Amyotrophic Lateral Sclerosis, Multiple Sclerosis) or non-neurological co-morbidities (e. g. heart failure, orthopaedic problems) |
| - Life expectancy < 1 year as determined by responsible physician |
| - Drug or alcohol addiction within the last six months |
| - Significant current psychiatric illness defined as medication-refractory of bipolar affective disorder, psychosis, schizophrenia or suicidality |
| - Current participation in another interventional trial |

supplementary Table 2 - Baseline characteristics diversified by extracellular vesicle type, (PV = platelet-derived vesicles, LV = leucocyte-derived vesicles, NV = neuronal-derived vesicles, EnV = endothelial-derived vesicles, MoV = monocytal-derived vesicles)

| Variable | Full cohort (n=110) | EnV high at baseline (n=27) | LV high at baseline (n=27) | MoV high at baseline (n=25) | NV high at baseline (n=27) | PV high at baseline (n=27) |
| --- | --- | --- | --- | --- | --- | --- |
| Age in years (median, IQR) | 69, 60 - 78.75 | 66, 53 – 76.5 | 66, 55 – 76 | 68, 54 - 76 | 68, 54 - 77 | 73, 56.5 - 80 |
| Sex (female, %) | 46, 43.39 | 13, 48 | 12, 44 | 11, 44 | 14, 52 | 12, 44 |
| NIHSS on admission (median, IQR) | 9, 6 – 12.75 | 10, 5 – 12 | 11, 5 – 11 | 11, 6 – 14 | 10, 5 – 12 | 9, 5.5 - 14 |
| Barthel Index on admission (median, IQR) | 50, 35 - 60 | 50, 45 – 62.5 | 50, 45 – 60 | 50, 40 – 55 | 55, 45 – 65 | 50, 45 - 60 |
